# Effect of Chronic L-Dopa or Melatonin Treatments after Dopamine Deafferentation in Rats: Dyskinesia, Motor Performance, and Cytological Analysis

**DOI:** 10.5402/2012/360379

**Published:** 2012-02-01

**Authors:** Ana Luisa Gutierrez-Valdez, Verónica Anaya-Martínez, José Luis Ordoñez-Librado, Ricardo García-Ruiz, Carmen Torres-Esquivel, Montserrat Moreno-Rivera, Javier Sánchez-Betancourt, Enrique Montiel-Flores, Maria Rosa Avila-Costa

**Affiliations:** Laboratorio de Neuromorfologia, Departamento de Neurociencias, Facultad de Estudios Superiores Iztacala, UNAM, Avenida de los Barrios 1, Los Reyes Iztacala, 54090 Tlalnepantla, MEX, Mexico

## Abstract

The present study examines the ability of melatonin to protect striatal dopaminergic loss induced by 6-OHDA in a rat model of Parkinson's disease, comparing the results with L-DOPA-treated rats. The drugs were administered orally daily for a month, their therapeutic or dyskinetic effects were assessed by means of abnormal involuntary movements (AIMs) and stepping ability. At the cellular level, the response was evaluated using tyrosine hydroxylase immunoreactivity and striatal ultrastructural changes to compare between L-DOPA-induced AIMs and Melatonin-treated rats. Our findings demonstrated that chronic oral administration of Melatonin improved the alterations caused by the neurotoxin 6-OHDA. Melatonin-treated animals perform better in the motor tasks and had no dyskinetic alterations compared to L-DOPA-treated group. At the cellular level, we found that Melatonin-treated rats showed more TH-positive neurons and their striatal ultrastructure was well preserved. Thus, Melatonin is a useful treatment to delay the cellular and behavioral alterations observed in Parkinson's disease.

## 1. Introduction

Parkinson disease (PD) is an age-related disorder characterized by a progressive degeneration of dopaminergic (DAergic) neurons of the substantia nigra pars compacta (SNc). The etiology of DAergic neurons death is not known. However, reported data suggest oxidative stress as the probable candidate to mediate in the original unknown cause. Studies on patients' brains have given evidences in support of this hypothesis. Levels of reduced glutathione (GSH) are low [1], while the level of the antioxidant enzyme manganese superoxide dismutase (SOD) is high and is not paralleled by a rise in glutathione peroxidase (GPX) [2]. Iron level increase has also been reported [3]. Since iron is able to catalyze the Fenton reaction, this implies hydroxyl radical (^•^OH) formation. Radical damage has been demonstrated in lipids [4], proteins, and nucleic acids [5] of the SNc of Parkinsonian patients. Thus, generation of reactive oxygen species (ROS) caused by oxidative stress, together with a relative lack of antioxidant defenses in the basal ganglia and nigrostriatal DAergic pathway, is commonly considered the final cause of neuronal death [6, 7].

L-DOPA (LD) is the drug of election in PD therapy. LD is converted by neuronal aromatic L-amino acid decarboxylase into dopamine (DA), reestablishing DA levels in surviving neurons, which, despite the treatment, continue to die [8, 9], and whereas LD treatment is very successful in the early stages of PD, it does not prevent disease progression, and in the long term there are undesirable side effects, such as the development of dyskinesias [10–12]. Dyskinesia interferes with physiological motor activity and constitutes a serious challenge to the management of PD (for review see Obeso et al. [13]). Previous studies have investigated the reasons for such long-term problems. Some suggested mechanism describes LD to elicit oxidative damage perpetuating the cell death [14–17], and, after MPTP infusion, it seems that LD generates 6-hydroxydopamine (6-OHDA) in the mouse striatum producing more ROS formation [18] enhancing iron-induced lipid peroxidation both *in vitro* and *in vivo* in the rat striatum [19]. Other evidences suggest that motor complications associated with sustained LD therapy are the consequence of irregular and intermittent delivery of LD to the brain, resulting in nonphysiologic pulsatile stimulation of striatal DA receptors [20]. Thus, the short half-life of immediate-release LD formulations is thought to be the key factor in the pathogenesis of motor complications [21]. Novel treatments for PD will be successful to the extent that they can either retard or prevent the development of these complications.

It has been recently established that DAergic and serotoninergic nuclei in the brain are overloaded with DA following acute, subacute, or chronic administration of LD [18], and the increased DA in the former nuclei can cause production of 6-OHDA in the brain [19]. Therefore, we hypothesized that Parkinsonian neurotoxins that generate ^•^OH in a DA-enriched environment would be conducive to oxidative stress production, and melatonin may act as a potent free radical scavenger and protect against ROS formation and the resulting DAergic neuronal death.

Melatonin is an indolamine first reported in 1993 by Tan et al. [22] as an efficient endogenous antioxidant. Melatonin possesses several unique advantages. First, its solubility in both lipids and water allows it to be easily distributed into the cell. Second, its ability to cross the brain-blood barrier allows melatonin to enter the central nervous system [23].

Melatonin has been proven to protect neuronal cells from neurotoxin-induced damage in a wide range of neuronal culture systems serving as experimental models for the study of PD (for review see [24]). *In vivo* experiments are however scarce and have almost always been done in acute experimental models of the disease. These acute studies show protective effects of melatonin in both the striatum DAergic axons [25] and the midbrain neurons [26].

There is considerable evidence that pharmacological doses of melatonin are neuroprotective in diverse models of neurodegeneration including PD. However, there is limited information about its effects on the initial stages of neurodegeneration.

Therefore, the present study was undertaken to determine the ability of melatonin to protect striatal DAergic loss induced by 6-OHDA in a rat model of PD, comparing the results with LD-treated rats. The drugs were administered four days following lesioning daily for a month at doses sufficient to improve physiological motor performance, and their therapeutic or dyskinetic effects were assessed using measures of abnormal involuntary movements (AIMs), skilled forelimb use, and stepping ability. At the cellular level, the response to the drugs was evaluated using tyrosine hydroxylase (TH) immunoreactivity and striatal ultrastructural changes to compare between LD-induced AIMs and melatonin-treated rats.

## 2. Experimental Procedures

The experiments were carried out in 24 male Wistar rats weighing 180–200 g at the beginning of the study. The rats were individually housed in hanging plastic cages under controlled light conditions (12 h light/h dark regime) and fed with Purina Rat Chow and water *ad libitum*. Body weight was recorded daily. The experimental protocol was conduced in accordance with the Animal Act of 1986 for Scientific Procedures. All efforts were made to minimize the number of animals used and their suffering.

### 2.1. Beam-Walking Task

Before 6-OHDA surgery, all animals were trained for one week to cross two narrow wooden beams (6 and 12 mm) into a safe platform. The beams measured 2 meters long and were elevated to a height of 1 m above the floor with wooden supports with 15° inclination. Each test session consisted of four trials in which latency to cross the beam was recorded (we established a maximum range of 120 seconds; if the animal did not cross at that time, the activity was terminated and we assigned the value of 120 seconds for that evaluation). Five trials were averaged to give a mean latency, and testing was done every week after 6-OHDA lesion. Training and testing were performed during the lighted portion of the cycle, at the same hour every time. Two observers blind to the rat treatment or control status perform all behavioral assessments.

### 2.2. Stereotactic Surgery and Treatments

The rats were anesthetized with sodium pentobarbitone (35 mg/kg i.p.) and placed in a stereotaxic apparatus. The rats were injected with 4 *μ*L of a saline solution containing 8 *μ*g of 6-OHDA (Sigma Chemical, USA) and 0.2 mg of ascorbic acid into the right medial forebrain bundle (*n* = 18), and sham lesion was made with vehicle (*n* = 6 (control group)). The injections were given over a 4 min period with a Hamilton syringe attached to a glass micropipette with a tip diameter of 20–50 *μ*m. The stereotaxic coordinates were as follows: AP = −4 mm anterior of the ear bar; *L* = 1.4 mm lateral of bregma; *V* = −7.7 mm vertical of dura (according to [27]). After recovery from the anesthesia, the animals were returned to their home cages. Apomorphine (Sigma Chemical, USA; 0.25 mg/kg i.p.) induced rotational behavior was tested two days after lesioning. Only those animals exhibiting more than 200 complete turns in a 30 min period were used [28]. Two days after the test, six experimental animals were treated with 7.5-mg/kg **L**-DOPA (Sinemet (Carbidopa-**L**-DOPA 25/250)), and 6 were treated with 10 mg/kg melatonin (Sigma Chemical, USA). The drugs were dissolved in 10 mL distilled water and given orally with an insulin syringe for a month. The other six lesioned rats without treatment, as well as the control animals, were kept for the same time. The motor performance was evaluated weekly.

### 2.3. AIMs Rating

LD-induced AIMs were scored at day 30 according to a rat dyskinesia scale [29–31]. Rats were placed individually in transparent plastic cages and observed every 20th min, from 20 min before to 180 min after giving LD (10 monitoring periods of 1 min each). Four subtypes of AIMs were classified according to their topographic distribution as locomotive, axial, forelimb, or orolingual (for details see Figure 4). Enhanced manifestations of otherwise normal behaviors, such as rearing, sniffing, grooming, and gnawing, were not included in the rating. AIM severity was assessed using the published method of Cenci et al. [29], Lee et al. [30], and Lundblad et al. [31], which assigns a score from 0 to 4 to each of the four AIM subtypes listed above according to the proportion of time/monitoring period during which the AIM is present. Borderline scores, such as 0.5, 1.5, 2.5, and 3.5, were allowed in order to increase the sensitivity of the evaluation.

### 2.4. Video Recording

Performance during beam walking test and AIM analysis was video recorded using a Panasonic camcorder (SDR-H80 model). Representative still frames were captured from digital video recordings with the video editing software Final Cut Pro. Pictures were cropped and adjusted for color and brightness contrast in Adobe Photoshop but were not altered in any other way.

### 2.5. Tissue Preparation

All rats were perfused under sodium pentobarbital anesthesia immediately after the one-month treatments via aorta, with saline solution followed by fixative containing 0.2% glutaraldehyde and 4% paraformaldehyde in 0.1 M phosphate buffer (PB). The brains were removed and placed in fixative solution for 1 hour.

### 2.6. TH Immunocytochemistry

Coronal sections (50 *μ*m) were obtained on a vibrating microtome through the mesencephalon for immunocytochemistry. Tyrosine hydroxylase (Chemicon International, Inc., CA, USA, 1 : 1000) immunostaining with the ABC detection method (Vector Lab MI, USA) was performed for light microscope analysis. The analysis was conducted with a computer-assisted system (Image-Pro Plus, Media Cybernetics, L.P. Del Mar, CA, USA) connected by a CCD camera to Optiphot 2 microscope (Nikon, Japan). The number of TH-positive neurons was counted in 1500 *μ*m^2^ from 7 SNc sections of each animal [32].

### 2.7. Electron Microscopy

Fragments from ipsilateral and contralateral striata were carefully taken. After washing in PB, the fragments were treated for 60 minutes with 1% osmium tetroxide in PB, washed for 30 minutes in PB, dehydrated with graded ethanol, and flat-embedded in araldite. Ultrathin sections were collected, counterstained with uranyl acetate and lead citrate, and examined in a JEOL 100CX-II electron microscope.

### 2.8. Ultrastructural Analysis

Synapses were defined by the presence of a clear postsynaptic density facing at least three presynaptic vesicles. Ultrastructural analysis was performed in 50 randomly selected synaptic endings per striatum. In each synaptic bouton, we observed all its membrane and organelle features, and we measured the following.

**Figure 1:**
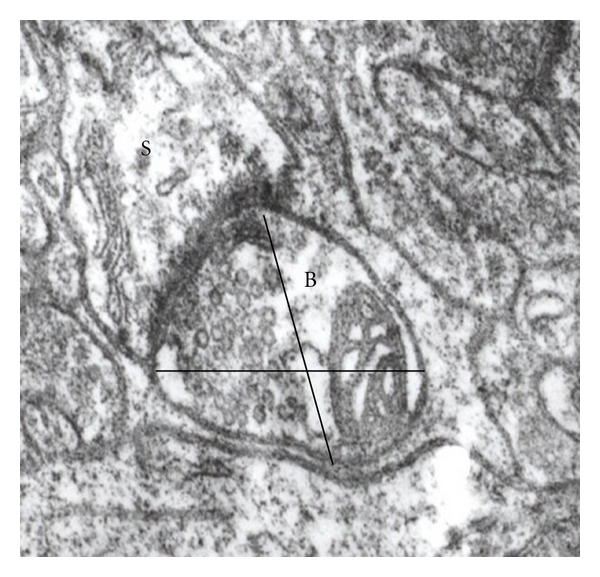
Synaptic ending (B) showing the two axes measured, establishing a synaptic contact with a dendritic spine (S).

- The diameter of the presynaptic bouton using two axes, which were perpendicular to each other and intersected at the center of the synaptic terminal (Figure 1); the diameter was measured directly from the electron microscope screen with a grid placed inside the eyepiece [33].
- The number of dendritic spines or dendrites as postsynaptic targets.
- Perforated synapses were identified on micrographs of serial sections and defined by the presence of a discontinuity in the postsynaptic density [34]. The number of perforated synapses was determined considering the following characteristics according to Calverley and Jones [35]: the site of the perforation projects into the presynaptic terminal, the active zone has one or more negatively curved components, which are separated by a central region of the active zone that projects into the presynaptic terminal, and the presynaptic density is in close association to the spine apparatus or an extension of it.

To minimize subjectivity, classification was carried out blind by at least two experimenters, and if distinction was unclear, the synapse was not included in the quantification.

### 2.9. Statistical Analysis

One-way ANOVA was used to analyze the number of TH-immunoreactive cells and behavioral data. Group differences were considered statistically significant at *P* < 0.05. When appropriate, *post hoc* comparisons were made with the Tukey test. All analyses were conducted with GraphPad Prism 5 Software.

## 3. Results

After 1 month, neither clinical alterations nor significant weight changes were detected in the experimental animals compared with controls.

### 3.1. Beam-Walking Test

**Figure 2:**
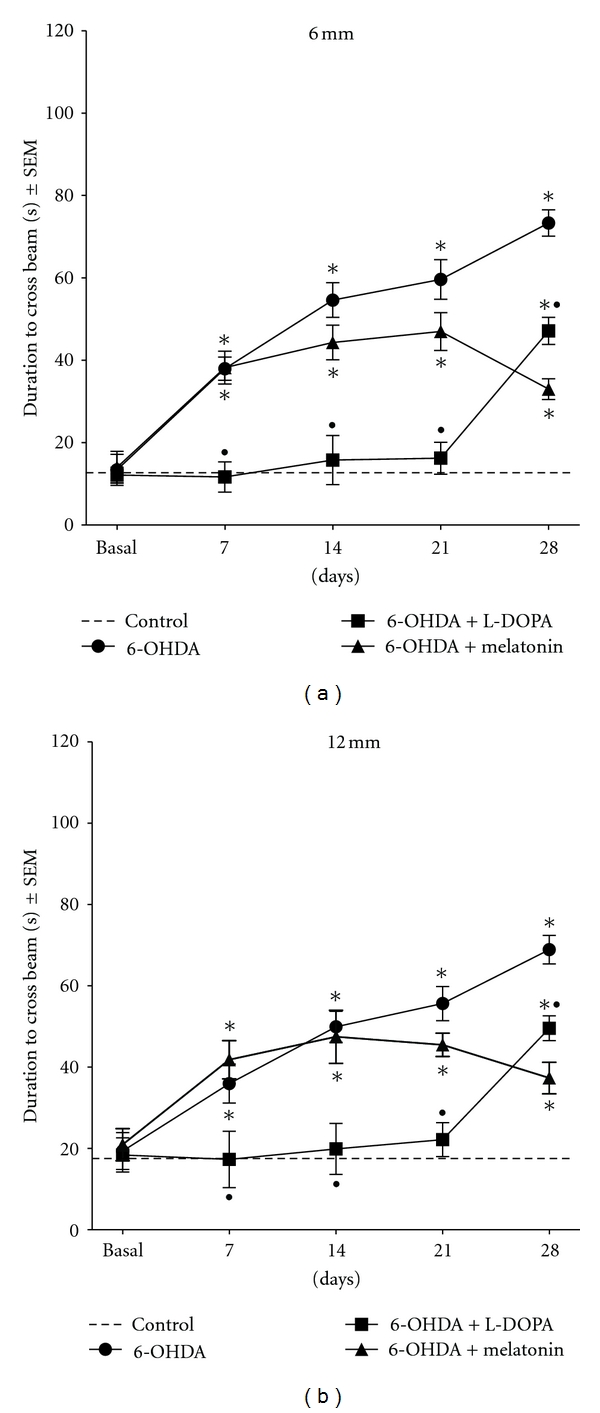
Mean latencies to cross two narrow beams (6 and 12 mm) (±SEM) before and after 6-OHDA lesion and during treatments. Note that from the beginning of LD treatment the rats improve their motor behavior until 21 days and afterwards showed a significant increase in the time to transverse the beam compared to controls. In contrast, the time to cross the beam of melatonin-treated rats until day 21th was similar to those animals with lesion and no treatment; afterwards the time was reduced drastically resembling the values of the control group (**^*∗*^** *P* < .001 versus control group; ^•^ *P* < .001 LD-treated group versus melatonin-treated group; ANOVA test).

Figure 2 illustrates the mean numbers of total time to cross the beam. In both beams 6 mm and 12 mm thick, control animals showed no significant difference in the time they took to traverse the beam. In contrast, 6-OHDA group, the animals had a very similar behavior in both beams, taking longer to traverse them, becoming more evident at more time after lesion; moreover lesioned animals with no treatment had some motor features such as freezing behavior, and, when moving, the rats were dedicated mainly to explore the beam rather to cross it, the rats steps were very slow compared to the control rats and several 6-OHDA-lesioned animals could not transverse the beam in the established time. On the other hand, the 6-OHDA-lesioned + LD treatment group since the beginning of treatment showed significant recovery of motor behavior until 21 days; these animals took less time to traverse the beams compared to the 6-OHDA-lesioned group and showed even better times than control group, suggesting hyperactivity, but afterwards had a significant increase in the time to cross and a significant potentiation of freeze time (data not shown) compared to control and 6-OHDA-lesioned + melatonin-treated rats. The 6-OHDA-lesioned + melatonin group at 7 and 14 days evaluation showed values very similar to 6-OHDA group without treatment, but, since 21 days, this group demonstrated an evident motor recovery.

### 3.2. Abnormal Involuntary Movements (AIMs)

#### 3.2.1. Time Course and Overall Incidence AIMs

**Figure 3:**
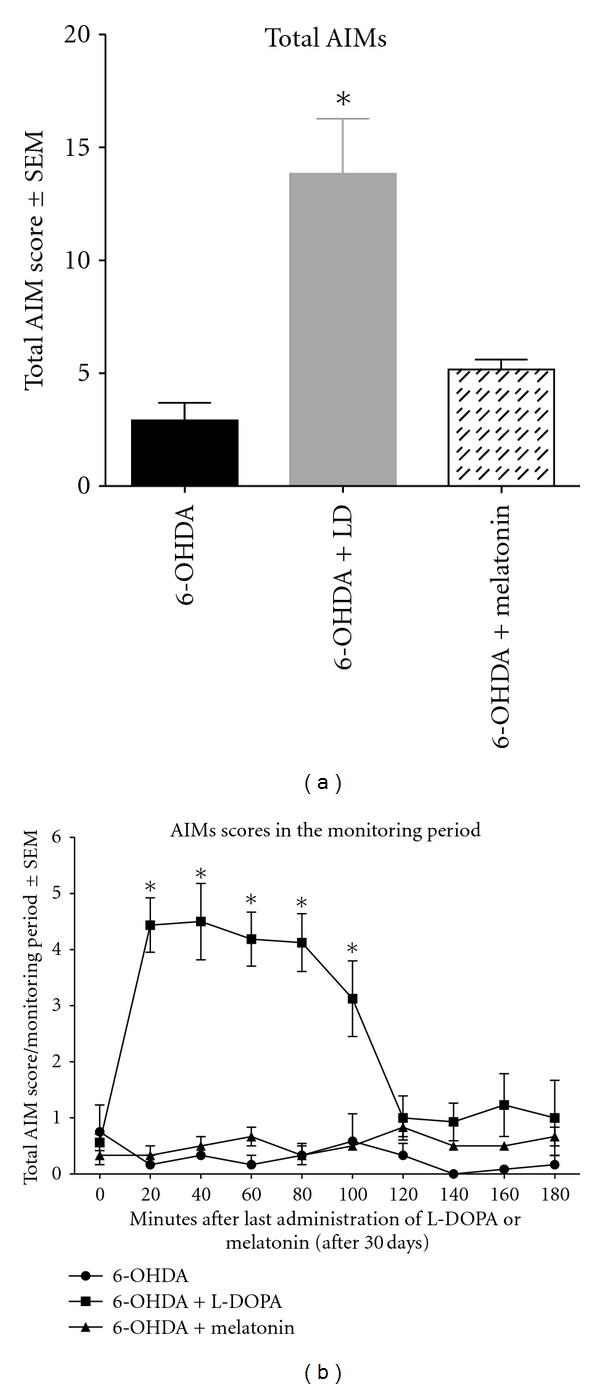
The three groups, 6-OHDA, 6-OHDA + LD, and 6-OHDA + melatonin confer certain susceptibility to dyskinesia during the course of the experiment, but the overall AIM severity is most pronounced in rats with 6-OHDA + LD treatment. (a) Time course of AIM development during the chronic LD and melatonin treatments period. Values give total (locomotive + axial + orolingual + limb AIMs) integrated AIM scores per testing session as group means ± SEM. (b) Time course of total AIM scores/monitoring period after a single treatment of LD or melatonin (treatment day 30).

In order to get an overview of the development of dyskinesia in the different groups, we carried out the summation of all subtypes of AIMs (axial + locomotive + limb + orolingual). As shown in Figure 3(a), repeated measures ANOVA revealed significant overall differences between 6-OHDA-lesioned and melatonin-treated groups comparing to LD-treated group.  Figure 3(b) (treatment day 30) depicts the temporal manifestation of AIMs after a dose of LD or melatonin resembling the time course of peak-dose dyskinesia in PD [36, 37]. Therefore, AIM severity progressively increased during the first 20 min posttreatment, continued elevated for an additional 60 min, and then gradually returned to baseline between 100 and 160 min posttreatment.

#### 3.2.2. Representation of AIM Subtypes

**Figure 4:**
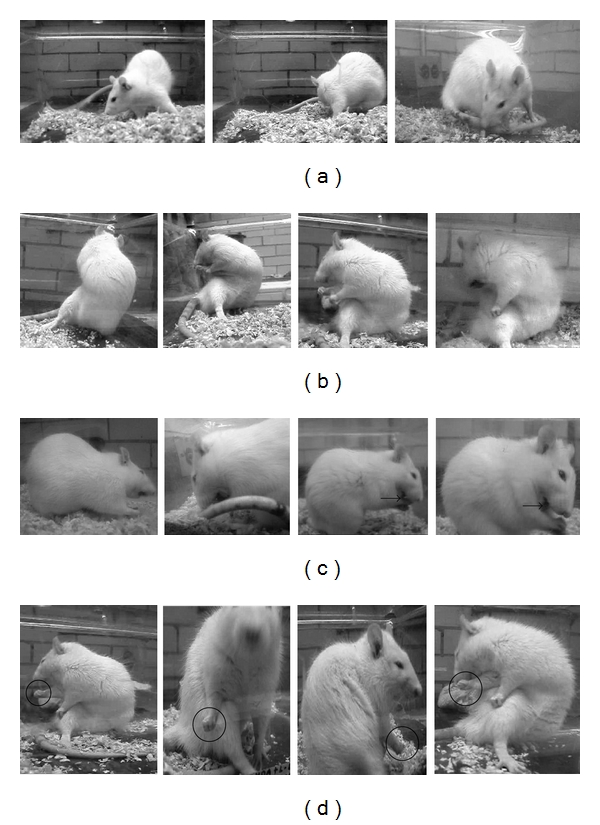
Sequences of video recordings from rats affected by locomotive (a), axial (b) orolingual (c), and forelimb AIMs (d). Locomotive AIMs (a) comprise circular movement towards the contralateral side to the lesion. Only locomotive movements involving all four limbs are rated under this AIM category. The sequence in (b) shows a torsion movement of the neck and upper trunk towards the contralateral side to the lesion. Body torsion is maximally severe (>90°), causing the rat to lose equilibrium. Orolingual AIMs (c) include opening and closing of the jaws and tongue protrusion towards the side contralateral to the lesion (arrow). A black circle in (d) highlights purposeless up and down translocation of the Parkinsonian (right) forelimb.

**Figure 5:**
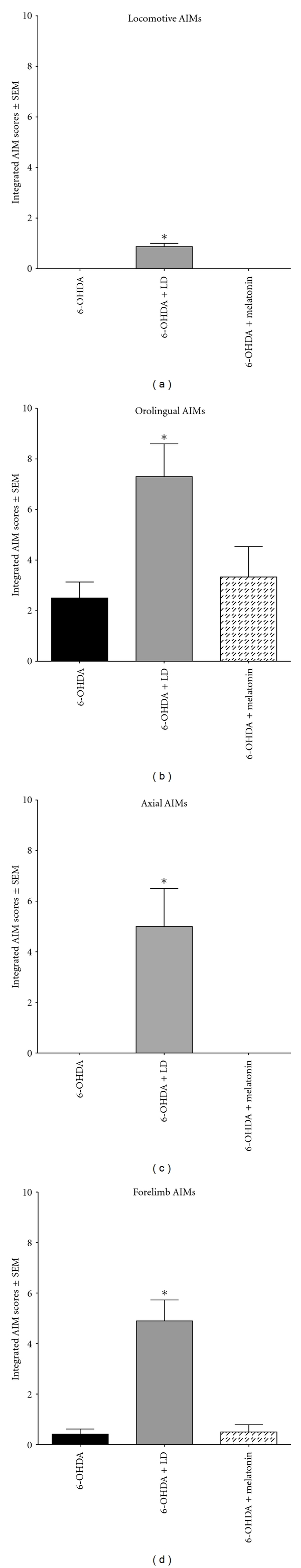
Integrated AIM scores were generated separately for locomotive (a), orolingual (b), axial (c), and forelimb (d) AIMs using data from day 30 of chronic treatments. Note that animals treated with melatonin did not develop locomotive AIMs and axial rotation (^*∗*^ *P* < 0.005 LD-treated group versus 6-OHDA-lesioned and melatonin-treated groups).

According to Cenci et al. [29] and Lee et al. [30], the animals were evaluated on four different topographic subtypes of AIMs, which are represented in Figure 4. Different AIM subtypes were mainly characterized among the LD-treated group (Figure 5). The 6-OHDA-lesioned animals and animals treated with melatonin showed no locomotive AIMs, contrasting to the animals treated with LD that manifest this behavior (Figure 5(a)). Animals treated with LD showed severely abnormal involuntary movements affecting the muscles of the neck and trunk, observed by the increase in axial AIMs score, unlike the 6-OHDA-lesioned animals and melatonin-treated group where these muscles were not affected (Figure 5(c)). Finally, the orolingual (Figure 5(b)) and forelimb AIMs (Figure 5(d)) were observed in all experimental groups; nevertheless, the score of the 6-OHDA-lesioned animals and melatonin-treated group were very small and showed no significant differences compared to control animals. However, animals treated with LD showed a significant increase in these dyskinetic movements.

### 3.3. TH Immunocytochemistry

**Figure 6:**
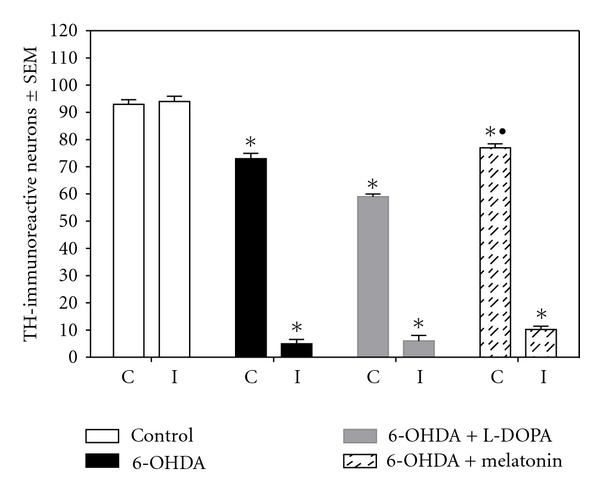
TH-immunoreactive cell counts from the SNc. The data are presented as the mean ± SEM. A statistically significant decrease in TH-immunoreactive cells was detected in both ipsilateral (I) and contralateral (C) SNc in the three experimental groups (^*∗*^ *P* < 0.05 versus control group; ^•^ *P* < 0.05 between melatonin and 6-OHDA and LD-treated groups; ANOVA test).

**Figure 7:**
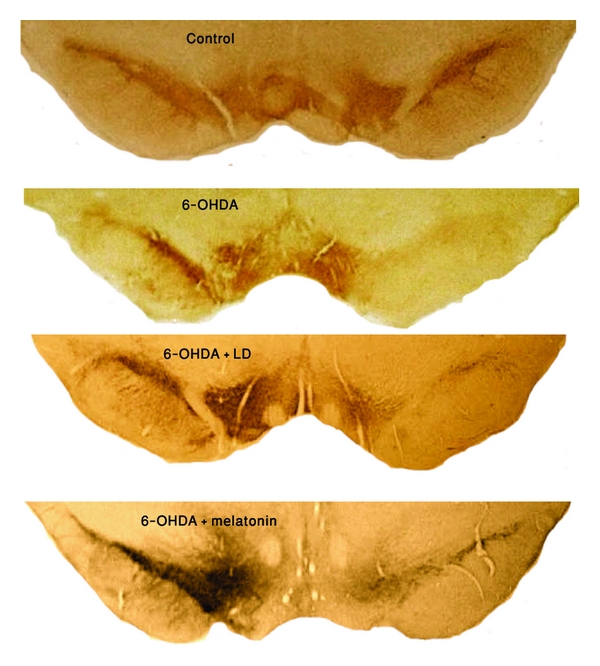
Representative TH-immunostained from coronal sections containing the SNc of control, 6-OHDA-lesioned, 6-OHDA-lesioned + LD and 6-OHDA-lesioned + melatonin-treated rats. Note the profound cell loss in the ipsilateral SNc in the three experimental groups, being more evident in the 6-OHDA and LD treated ones; also, the contralateral SNc of melatonin-treated rats lost fewer neurons than the other two experimental groups (magnification 4×).

The number of TH-positive neurons in the control group, both the ipsi and contralateral SNc remained unchanged (*x* = 93 ± 1.7 and *x* = 94 ± 1.9, resp.). In contrast, we found an important loss of TH-positive neurons in the SNc of 6-OHDA lesioned animals in both ipsilateral (*x* = 5 ± 1.6) and contralateral (*x* = 73 ± 1.9) SNc compared to controls as shown in Figures 6 and 7; likewise, LD-treated rats (*x* = 59 ± 1.0 and 6 ± 2.0 contralateral and ipsilateral, resp.) and melatonin-treated rats (*x* = 77 ± 0.48 contralateral and *x* = 10.2 ± 0.218 ipsilateral SNc) show significant loss of TH-positive cells; however, melatonin-treated animals exhibited less neuronal loss compared to the group treated with LD.

### 3.4. Electron Microscopy

#### 3.4.1. Diameters of Synaptic Endings

**Figure 8:**
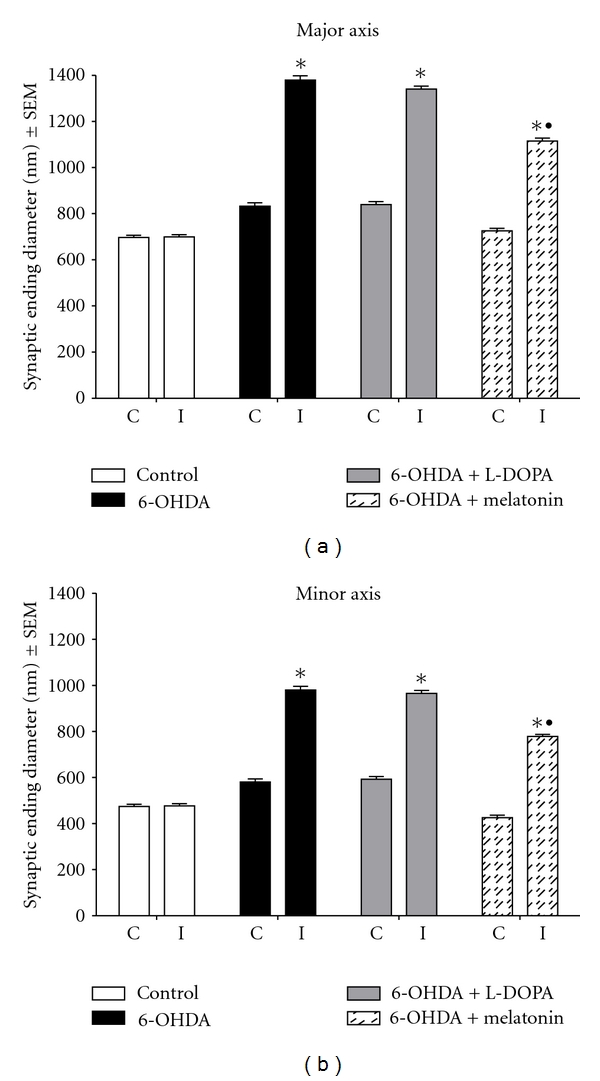
Synaptic ending mean diameter in ipsilateral (I) and contralateral (C) striata after stereotactic surgery and treatments, major and minor axes (^*∗*^ *P* < 0.05 versus control group; ^•^ *P* < 0.05 between melatonin and 6-OHDA and LD-treated groups; ANOVA test).

**Figure 9:**
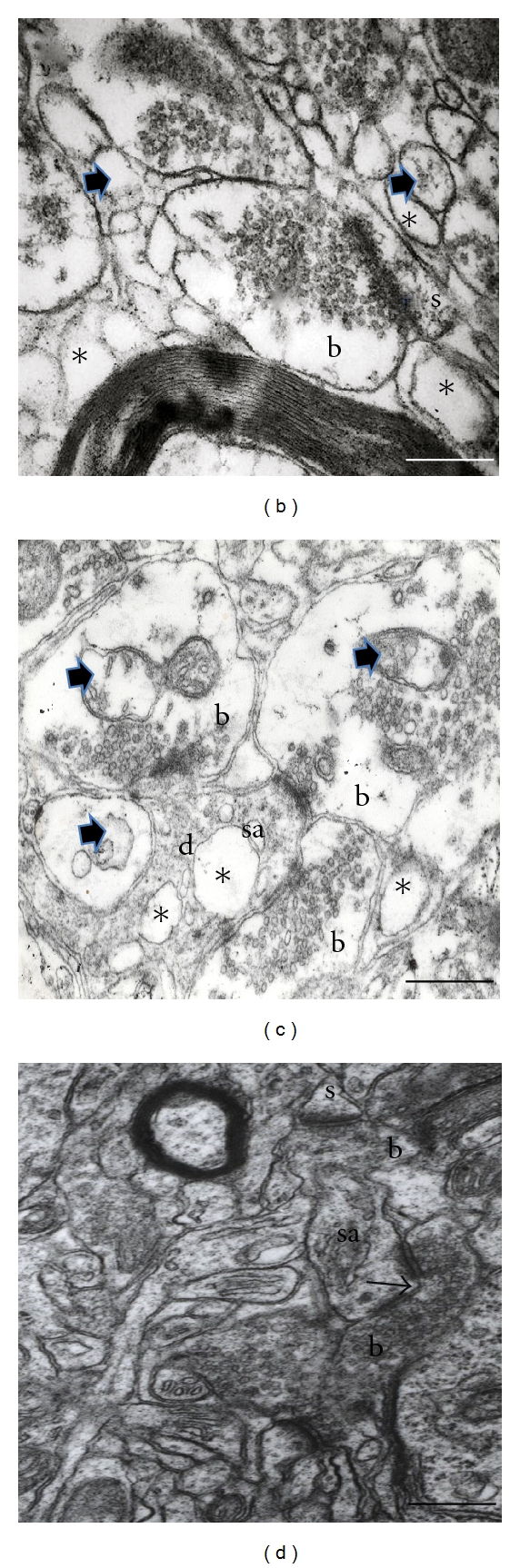
Electron micrographs from control group rat striatum neuropile (A); 6-OHDA-lesioned ipsilateral striatum (B); 6-OHDA + LD treated rat ipsilateral striatum (C); 6-OHDA + melatonin-treated rat ipsilateral striatum (D). (A) In control group, the mean size of the synaptic buttons (b) was 700 × 696 nm, and the predominant Postsynaptic target was the dendritic spines (s); it can be observed that the neuropile is well preserved. (B) This image shows a swollen synaptic button (b) establishing a synaptic contact with a dendritic spine (s), altered mitochondria (arrowhead), and some vacuoles (*) within neuropile. (C) This image demonstrates three edematous presynaptic endings (b) of the LD-treated ipsilateral striatum establishing three synaptic contacts, one with a dendritic spine with dilated spine apparatus (sa), and two with a dendrite (d). Note the altered mitochondria (arrow heads) and neuropile vacuoles (*). (D) An increase in the presence of perforated synaptic contacts was notorious in striata of the three experimental groups (arrow). Note that the neuropile of the melatonin-treated group is well preserved, similar to control group neuropile. (b) Synaptic bouton, (sa) spine apparatus, (s) dendritic spine. Bar 0.2 *μ*m.

Control rats did not show any differences between both striata synaptic endings diameter and neuropile alterations after sham surgery (Figures 8 and 9(A)). As shown in Figure 8, the synaptic endings of the control group major axis presented an average of 696.8 ± 9.4 nm on the contralateral striatum and 700 ± 9.6 nm on the ipsilateral one; the minor axis mean was 474.9 ± 9.6 nm on the contralateral and 477.0 + 9.6 *μ*m^2^ on the ipsilateral striatum. The 6-OHDA-lesioned group showed an evident increase in the size of synaptic boutons (*x* = 980.3 ± 16.13 and 1379.7 ± 18 nm minor and major axis, respectively, of the ipsilateral striatum); the same pattern was observed in the 6-OHDA + LD-treated group (*x* = 966.0 ± 12.10 and 1340.0 ± 13.20 nm minor axis and major axis of the ipsilateral side, resp.); there were statistically significant differences in both groups comparing to control group (Figure 8). 6-OHDA + melatonin-treated group showed fewer presynaptic buttons with edema (*x* = 778.7 ± 11.05 nm and 1115.30 ± 11.00 nm minor and major axis of the ipsilateral side, resp.), showing statistically significant differences comparing to 6-OHDA + LD and lesioned untreated groups. The contralateral (nonlesioned side) of all experimental groups (6-OHDA, 6-OHDA + L-DOPA and 6-OHDA + melatonin) showed no statistically significant differences compared to control group (Figure 8).

#### 3.4.2. Postsynaptic Target

**Figure 10:**
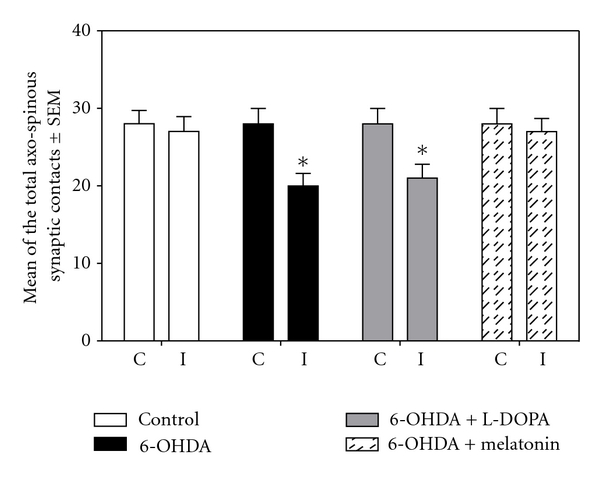
This graph shows the average number of synaptic boutons that established synaptic contact with dendritic spine in the ipsilateral and contralateral striata of the four analyzed groups (*P < 0.005 versus control group and melatonin-treated group; ANOVA test).

When analyzing the postsynaptic structure (spine or dendrite) with which synaptic contact was established, we note that in control group prevailed synaptic contacts with dendritic spines (Figures 9(A) and 10) show an average of 28 ± 1.0 for the contralateral side and 27 ± 1.9 for the ipsilateral one, unlike 6-OHDA group (*x* = 20 ± 1.6) and 6-OHDA + LD-treated group (*x* = 21 ± 1.8), where there was an important decrease in the number of synaptic contacts established with dendritic spines in ipsilateral striatum (Figures 9(B), 9(C), and 10); there were significant differences comparing to control group and the group treated with melatonin; the latter presented similar number of synaptic contacts with dendritic spines than the control group (Figures 9(D) and 10).

#### 3.4.3. Perforated Synapses

**Figure 11:**
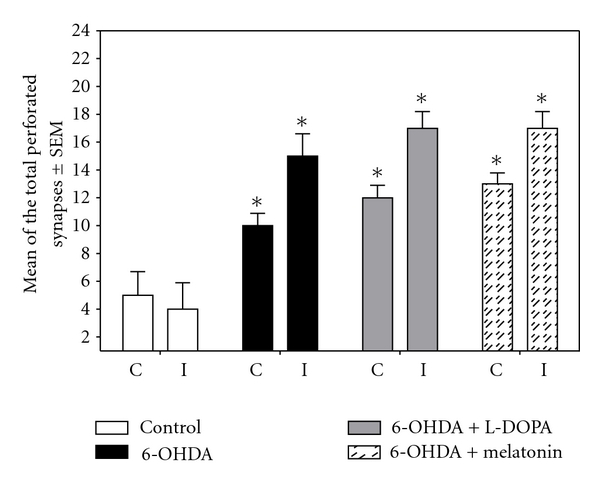
This graph shows the average of the total number of perforated synapses in the ipsilateral and contralateral striata of the four analyzed groups (*P < 0.005 versus control group and melatonin-treated group; ANOVA test).

Figure 11 depicts the number of perforated synaptic contacts in the different groups; the control group had a mean of 5 ± 1.7 contralateral and 4 ± 1.9 in the ipsilateral striata, in contrast; all experimental groups showed an increase in the number of perforated contacts in both striata, becoming more evident in the ipsilateral side; we found significant differences compared to the control group (Figures 9(D) and 11).

## 4. Discussion

The findings of the present study demonstrated that chronic oral administration of melatonin corrected a hemi-Parkinson's condition in rats caused by intramedial forebrain bundle application of the neurotoxin 6-OHDA. melatonin-treated animals perform better in the motor tasks and had no dyskinetic alterations compared to LD-treated rats. At the cellular level, we found that melatonin-treated rats showed more TH-positive neurons and their striatal ultrastructure was well preserved, which probably led to the functional recovery.

### 4.1. Motor Behavior

Our results show that 6-OHDA-lesioned animals displayed decreased motor coordination, becoming more evident over time following lesion. This finding is consistent with the results of Allbut and Henderson [38], who demonstrate that rats unilaterally lesioned with 6-OHDA in the medial forebrain bundle presented motor behavior alterations when evaluated on a beam-walking test; they observed that the time the rats took to traverse the beam was drastically increased compared to control group; animals also showed rigidity and restraint in their movements when walking the beam. Some rats roamed the beam and stopped and then restarted or just stand still, which is analogous to the behaviors observed in patients with PD. Also, Truong et al. [39] conducted a study in rats unilaterally lesioned with 6-OHDA at different doses and subsequently evaluated motor behavior in a wooden beam. The animals showed a travel time of about 1 minute at 2 and 4 weeks after lesion, while the control group showed a travel time of 0.2 minutes, concluding that changes in motor performance are correlated with the loss of DAergic cells.

As described in our results, animal treated with LD showed improvement to traverse the beam until 21 days, but after 28 days the treatment was no longer able to reduce the motor alterations induced by 6-OHDA-lesion Our results also show that animals with DA denervation reproduce dyskinetic motor effects when treated with therapeutical doses of LD. As we mentioned previously, LD treatment is the most effective drug for PD treatment, since no other drug matches its ability to suppress the symptoms. However, after chronic treatment, 30 to 80% of patients develop side effects such as dyskinesias that become more disabling than the disease itself [40]. In this regard Rajput et al. [41] stand out in their review that patients who received LD, about two-thirds, had benefits within the first three to six months of treatment, but after a while show a clear deterioration accompanied by a constant increased complications, such as changes in threshold and AIMs [42]. It has also been reported that patients who develop dyskinesias due to prolonged use of LD are characterized by involuntary and uncontrollable chaotic movements of the mouth, cheeks, arms, and legs [43, 44]; these dyskinesias may be considered a negative reaction of brain plasticity in response to the time of disease progression and prolonged use of LD [44, 45].

The mechanism by which LD induces motor complications has not been established, but several theories have been proposed: (A) the fluctuations may occur due to deficiencies in the synthesis and storage of DA in DAergic terminals [40]; (B) the DA transporter system, which represents a major mechanism by which DA is removed from the synapse (which is a crucial element involved in the regulation of DAergic transmission) [46], is altered by prolonged LD treatment [47]; (C) it seems that LD produce oxidative damage perpetuating cell death [14–17] enhancing iron-induced lipid peroxidation [19]. Other evidences suggest that motor complications associated with sustained LD therapy are a result of irregular and intermittent delivery of LD to the brain, resulting in nonphysiologic pulsatile stimulation of striatal dopamine receptors [20].

On the other hand, animals treated with melatonin showed a motor performance very similar to 6-OHDA-lesioned group at 7 to 14 days; afterwards the animals improved; this became more evident at 28 days after lesion. We consider that melatonin-treated animals probably do not improve their motor behavior at the start of treatment, as the neurotoxin is highly aggressive and causes some cells no longer functional (i.e., discontinue producing DA) without necessarily inducing death [48], and therefore display a motor imbalance very similar to 6-OHDA-lesioned group; thus it is feasible to think that melatonin treatment trigger different signaling pathways to enhance the mechanism against ROS produced by 6-OHDA; so after some time it is possible that the cell is able to recover its DAergic phenotype, producing a modulatory effect reflected in functional recovery. It has also been reported that elevated ROS participate in 6-OHDA neurotoxicity; this has been evidenced by the reduction in brain GSH levels and in the loss of SOD activity [49]. Melatonin stimulates antioxidant enzymes such as SOD, GPX, and glutathione reductase [50]. Singh et al. [51] demonstrated that systemic administration of melatonin protected striatal DAergic neurons against 6-OHDA neurotoxicity in the rat. The effect was accompanied by a significant recovery in motor behavior tests.

In this regard Singh et al. [51] have reported that animals pretreated systemically with melatonin and subsequently lesioned with 6-OHDA and treated with melatonin for a period of 7 days showed a decrease in the number of apomorphine-induced rotations, improved posture, and slowness of movement compared to 6-OHDA-lesioned treated with vehicle solution group. These results demonstrate that melatonin treatment may have beneficial effects for the treatment of PD. Furthermore, Hamdi [52] conducted a study in animals where he administered melatonin in drinking water and found that the striatum had a higher affinity to D2 receptor, while the number of receptors did not change. The mechanism by which melatonin increases the affinity of D2 receptors is unknown, but the author suggests that this effect may be produced through conformational changes in the receptor-binding site. This mechanism may involve a functional alteration by a direct or indirect effect of melatonin on one or more levels that is, gene expression and receptor proteins synthesis; thus melatonin may represent a modulatory influence on the DAergic system [53].

### 4.2. TH Immunocytochemistry

We observed a severe decrease of TH-positive cells after depleting the nigrostriatal pathway; our results agree with Damier et al. [54] who found that PD patients showed a reduction of DAergic neurons up to 95% depending on the time of clinical evolution. Likewise, it has been demonstrated that the medial forebrain bundle unilateral lesion reduces 98% the SNc ipsilateral number of TH-immunoreactive neurons [38, 55]. We found that 6-OHDA-lesioned and 6-OHDA + LD-treated groups displayed a very similar pattern, showing a dramatic loss of TH-immunoreactive neurons. The cell loss after LD treatment may have been because LD is converted to DA by the enzyme AADC and thus raises the levels of DA in the striatum [56], this in consequence may act as a cell death perpetrator, since there is evidence that suggests that large amounts of DA, in addition to serving as a neurotransmitter, can act as a neurotoxin. It has been found in culture cells exposed to high levels of DA that DA is able to produce apoptotic neuronal death [57], decrease levels of GSH, and increase intracellular Ca^2+^; these effects of DA oxidation in cellular physiology *in vivo* may occur under conditions of oxidative stress, increasing the vulnerability of DAergic neurons to degeneration [58]. Furthermore, Maharaj et al. [19] demonstrate that LD might accelerate the rate of SNc degeneration because it undergoes oxidative metabolism to form 6-OHDA.

About the data obtained after melatonin treatment, we observed that this indolamine partially prevents SNc DAergic cell damage produced by 6-OHDA lesion. 6-OHDA toxicity is based on direct inhibition of complex I of the electron transport chain in the mitochondria [59]. Inhibition of this complex has also been reported in the SNc of patients suffering PD. This inhibition causes energy depletion and increases free radical concentration in the mitochondria [60]. In the present work, for melatonin-treated group, although the cell percentage decreases (comparing ipsilateral to contralateral sides), the loss was less severe than that detected in 6-OHDA-lesioned and LD-treated rats. The antioxidant effects of melatonin and its protective effects against the uncoupling of the electron transport chain of several toxins in the mitochondria have been well summarized by Acuña-Castroviejo et al. [61]. These data urge us to undertake further studies based on this hypothesis. *In vivo* studies with melatonin in experimental models of PD are however scarce. Acuña-Castroviejo et al. [25] found that melatonin prevented an increase in lipid peroxidation and a decrease in TH immunoreactivity in the striatum after a single dose of MPTP, arguing that melatonin was able to prevent the damage caused by this drug in the striatal dopaminergic axons. Ortiz et al. [26] reported, using DNA electrophoresis, apoptosis of midbrain neurons induced by a single dose of MPTP. Melatonin also prevented both cell damage and DNA fragmentation. Also, melatonin was able to counteract the decrease in striatal TH immunoreactivity and the loss of complex I activity produced in rats after acute 6-OHDA administration [62, 63]. Similarly, Kim et al. [64] showed that melatonin treatment rescues nigrostriatal dopaminergic neurons from cell death in 6-OHDA-lesioned rats suggesting that this beneficial effect was a consequence of the potent antioxidative action of melatonin. Thus, melatonin has been reported to protect against 6-OHDA [63], MPTP [25], and MPP^+^ lesions [65]. However, it should be emphasized that these melatonin-induced effects have been traditionally discussed in the context of the efficient free radical scavenger and antioxidant properties of this pineal hormone (for a review see Reiter [24]). Nevertheless, our data show a stimulating effect of melatonin over TH-immunoreactivity suggesting a role over the nigrostriatal DAergic system. In fact, melatonin has been shown to regulate striatal dopaminergic activity and block LD-induced dyskinesias [66]. As we mentioned above, it is therefore plausible to assume a role for melatonin as a neuromodulator in the nigrostriatal DAergic system [67]; however, further analyses are needed to clarify this point.

### 4.3. Electron Microscopy

The ultrastructural analysis of the striatum neuropile after unilateral 6-OHDA lesion of the medial forebrain bundle of rats and after LD treatment revealed that this neurotoxin induces derangement of the ipsilateral striatum neuropile characterized by (1) edema of the presynaptic buttons, (2) changes on the postsynaptic targets, and (3) increase in the number of perforated synapses, alterations that improved with melatonin treatment.

The present analysis confirms previous observations [33, 68–70], concerning to the fact that DA depletion of the nigrostriatal pathway causes an increment in the size of synaptic buttons. The authors assume that this swelling is due to an inherent degenerative process caused by 6-OHDA lesion, because they saw this increment in size in almost all synaptic endings that were measured. Here, we observe that melatonin treatment prevented synaptic ending swelling. Biochemical abnormalities relevant to the pathogenesis of PD include mitochondrial dysfunction, free radical-mediated damage, excitotoxicity, and inflammation [71, 72]. About the anti-inflammatory effects of melatonin, the most important feature is its inhibition of iNOS expression [73]. melatonin administration prevented mitochondrial iNOS induction in sepsis and in MPTP-treated mice, avoiding respiratory chain dysfunction and preserving ATP production [61, 74–76]. The indolamine also increases the activity of the respiratory complexes, counteracts the oxidative stress, and maintains the mitochondrial GSH pool under different experimental conditions both *in vivo* and *in vitro* [77]. These antioxidant and anti-inflammatory properties of melatonin are relevant in mitochondrial physiology, and they may play a neuroprotective role in PD [71].

Concerning dendritic spine loss, Roberts and DiFiglia [78], Ingham et al. [68], Pickel et al. [69], Stephens et al. [79], and Avila-Costa et al. [33, 70] found that the proportion of axospinous synapses was significantly reduced in the ipsilateral striatum of the 6-OHDA lesioned rats, type of synapse that also decrease in caudate nucleus of PD patients [80–82]. Here we found an evident loss of dendritic spines in 6OHDA-lesioned and LD-treated rats. Schuster et al. [83] reported that, after 6-OHDA lesion and LD treatment, the rats presented a marked loss of dendritic spines in the lesioned striatum, suggesting that the prevention of dendritic spine loss is crucial to impede AIMs. However, it is possible that the role for spine loss after DA depletion could be adaptive and might prevent degeneration of striatal neurons from a sustained and excessive glutamatergic input in the absence of endogenous DAergic control of the excitatory transmission [83]. There is no doubt that the spine loss response is adaptive because preventing the elevation in dendritic excitability significantly reduces spine loss [84, 85]. Nevertheless, melatonin treatment, here, prevented the dendritic spine loss, a fact that is related to the greater number of TH-positive cells and the functional recovery, making melatonin a plausible candidate to avoid disease progression.

On the other hand, previous reports stand out that nigrostriatal synaptic terminals most commonly form contacts with dendritic spines and less commonly with the somata or dendrites of striatal neurons [86–91]. Following 6-OHDA lesioning, the number of distal dendrite and spine contacts decrease, and consequently there are a greater proportion of more proximal dendrite and soma contacts [91, 92]. More recently, Reynolds et al. [93] described how stimulation of the SNc induced potentiation of the glutamatergic synapses between the cortex and the striatum that was dependent on activation of DA receptors. The corticostriatal glutamatergic fibres synapse onto the heads of dendritic spines of the striatal neurons, whereas the SNc terminals normally synapse onto the spine necks. As more proximal synapses are believed to elicit greater physiological changes in the target neurons than distal synapses [69], the more proximal site of termination of the reinnervated DA terminals could enhance the efficiency of DA intensification of glutamatergic transmission. Indeed, Picconi [94] described that plasticity at the cortical projection onto spiny neurons was altered by selective DA receptor blockade and following DA denervation but restored by LD therapy, at least at the start of treatment [94–96]. Other authors have noted that, following neuroleptic treatment, there is persistent modification in dendrites and spines, especially in the ventral striatum. As lesioning and LD therapy both produce damage [97], it is possible that this alteration provides the drive for the synaptic remodeling described here and elsewhere [98–100], such as the increment in the number of perforated synapses [34]. We speculate that the altered morphology and function of these terminals not only reflect mechanisms that may compensate for the loss of nigral neurons but may also be important in understanding the molecular processes underlying the dyskinesia induced by LD treatment.

Finally, the incremented number of perforated synapses in the three experimental groups is in agreement with other authors who reported an important proliferation of perforated synapses following harming circumstances, hippocampal kindling [101], unilateral lesion of the entorhinal cortex [102], * *and other brain lesions [103]. These authors suggest that the perforations may function to increase the perimeter surface of the postsynaptic density and also the efficiency of neurotransmission. Furthermore, this kind of synapses are intermediate structures in an ongoing cycle of the breakdown and replacement of synapses that are lost because of a specific lesion, transmission changes, or ageing process [104]. In this way, See et al. [105] demonstrate that after chronic neuroleptic treatment, which increases the DAergic binding sites, there was a significant increment in the number of perforated synapses. In that report, the authors found an important proliferation in perforated synapses after the lesion, maybe as a consequence of the postsynaptic supersensitivity of the DAergic receptors reported by Ungerstedt [106], which in turn induces synaptic plasticity, increasing the length of the postsynaptic density [34]. Therefore, selective synaptic changes in shape and function are possibly signs of excitotoxic injury, as observed in diverse neurological diseases and neurodegenerative disorders. Elucidating mechanisms that mediate the synaptic alterations under pathological conditions may be of fundamental importance to understanding mechanisms of neuronal injury. In spite of many new findings, there are still various questions to be answered and further experiments to be done. The mechanisms of synaptic plasticity are still not completely clear: the role of retrograde messengers, details in the molecular cascades leading to gene expression and new protein synthesis or to growth of new synapses, finding the more accurate causal connection between plasticity and various forms of learning, memory, and cell. The use of regulated and anatomically restricted genetic modification, combined with morphologic analysis, should provide a powerful set of tools for elucidating synaptic plasticity mechanisms damage [107].

In conclusion, the data described in the present study provides further evidence that melatonin acts as a DA regulator impeding partially the DAergic cell death by means of preservation of the striatal neuropile and the dendritic spines, promoting functional recovery, since melatonin-treated rats displayed better motor performance and no dyskinetic alterations, compared to LD-treated rats.

Melatonin displays an important antioxidant property based on its ability to function as a free radical scavenge. Particularly, in contrast to conventional antioxidants, melatonin can rapidly cross the blood-brain barrier after systemic administration. This unique character enables melatonin to directly reach the neuronal compartment [23]. At this point, we have to focus on early, long-term, preventive administration of melatonin. Based on previous and current results, melatonin is more likely the complementary and alternative therapy.

It is important to stand out that levels of melatonin tend to decrease with age in contrast to the increased incidence of neurodegenerative diseases. Aging and neurodegenerative diseases have been proposed as a consequence of the imbalance (physiological or toxin-induced) between oxidant production by the organism and its antioxidant defense system. Other constituents of this antioxidant system have not been found to decrease with age, melatonin being the only one matching this age-related pattern. This points to an increase in free radicals which are endogenously or exogenously produced. The protective effect of melatonin demonstrated in abundant cell culture experiments, together with the *in vivo* protection against 6-OHDA and MPTP-induced cell damage, makes melatonin a plausible candidate in the prevention of the appearance of these diseases and gives a clue to its use as a treatment to avoid disease progression [60]. Consequently, melatonin administration combines antioxidant capacity along with a tissue-specific TH-inducing effect, which could be beneficial for treating PD.

## References

[1] Perry TL, Yong VW (1986). Idiopathic Parkinson’s disease, progressive supranuclear palsy and glutathione metabolism in the substantia nigra of patients. *Neuroscience Letters*.

[2] Saggu H, Cooksey J, Dexter D (1989). A selective increase in particulate superoxide dismutase activity in parkinsonian substantia nigra. *Journal of Neurochemistry*.

[3] Dexter DT, Wells FR, Lees AJ (1989). Increased nigral iron content and alterations in other metal ions occurring in brain in Parkinson’s disease. *Journal of Neurochemistry*.

[4] Yoritaka A, Hattori N, Uchida K, Tanaka M, Stadtman ER, Mizuno Y (1996). Immunohistochemical detection of 4-hydroxynonenal protein adducts in Parkinson disease. *Proceedings of the National Academy of Sciences of the United States of America*.

[5] Alam ZI, Daniel SE, Lees AJ, Marsden DC, Jenner P, Halliwell B (1997). A generalised increase in protein carbonyls in the brain in Parkinson’s but not incidental Lewy body disease. *Journal of Neurochemistry*.

[6] Coyle JT, Puttfarcken P (1993). Oxidative stress, glutamate, and neurodegenerative disorders. *Science*.

[7] Ebadi M, Srinivasan SK, Baxi MD (1996). Oxidative stress and antioxidant therapy in Parkinson’s disease. *Progress in Neurobiology*.

[8] Basma AN, Morris EJ, Nicklas WJ, Geller HM (1995). L-DOPA cytotoxicity to PC12 cells in culture is via its autoxidation. *Journal of Neurochemistry*.

[9] Corona-Morales AA, Castell A, Escobar A, Drucker-Colín R, Zhang L (2003). Fullerene C60 and ascorbic acid protect cultured chromaffin cells against levodopa toxicity. *Journal of Neuroscience Research*.

[10] Ahlskog JE, Muenter MD (2001). Frequency of levodopa-related dyskinesias and motor fluctuations as estimated from the cumulative literature. *Movement Disorders*.

[11] Jankovic J (2005). Motor fluctuations and dyskinesias in Parkinson’s disease: clinical manifestations. *Movement Disorders*.

[12] Schrag A, Ben-Shlomo Y, Quinn N (2002). How common are complications of Parkinson’s disease?. *Journal of Neurology*.

[13] Obeso JA, Olanow CW, Nutt JG (2000). Levodopa motor complications in Parkinson’s disease. *Trends in Neurosciences*.

[14] Miller JW, Selhub J, Joseph JA (1996). Oxidative damage caused by free radicals produced during catecholamine autoxidation: protective effects of O-methylation and melatonin. *Free Radical Biology and Medicine*.

[15] Olanow CW, Agid Y, Mizuno Y (2004). Levodopa in the treatment of Parkinson’s disease: current controversies. *Movement Disorders*.

[16] Lee M, Tazzari V, Giustarini D (2010). Effects of hydrogen sulfide-releasing L-DOPA derivatives on glial activation: potential for treating Parkinson disease. *Journal of Biological Chemistry*.

[17] Milusheva E, Baranyi M, Kormos E, Hracskó Z, Sylvester Vizi E, Sperlágh B (2010). The effect of antiparkinsonian drugs on oxidative stress induced pathological [3H]dopamine efflux after in vitro rotenone exposure in rat striatal slices. *Neuropharmacology*.

[18] Borah A, Mohanakumar KP (2009). Melatonin inhibits 6-hydroxydopamine production in the brain to protect against experimental parkinsonism in rodents. *Journal of Pineal Research*.

[19] Maharaj H, Sukhdev Maharaj D, Scheepers M, Mokokong R, Daya S (2005). L-DOPA administration enhances 6-hydroxydopamine generation. *Brain Research*.

[20] Poewe W, Antonini A, Zijlmans JC, Burkhard PR, Vingerhoets F (2010). Levodopa in the treatment of Parkinson's disease: an old drug still going strong. *Journal of Clinical Interventions in Aging*.

[21] Olanow CW (2004). The scientific basis for the current treatment of Parkinson’s disease. *Annual Review of Medicine*.

[22] Tan DX, Manchester LC, Reiter RJ (1998). A novel melatonin metabolite, cyclic 3-hydroxymelatonin: a biomarker of in vivo hydroxyl radical generation. *Biochemical and Biophysical Research Communications*.

[23] Reiter RJ, Cabrera J, Sainz RM, Mayo JC, Manchester LC, Tan DX (1999). Melatonin as a pharmacological agent against neuronal loss in experimental models of Huntington’s disease, Alzheimer’s disease and Parkinsonism. *Annals of the New York Academy of Sciences*.

[24] Reiter RJ (1998). Oxidative damage in the central nervous system: protection by melatonin. *Progress in Neurobiology*.

[25] Acuña-Castroviejo D, Coto-Montes A, Monti MG, Ortiz GG, Reiter RJ (1996). Melatonin is protective against MPTP-induced striatal and hippocampal lesions. *Life Sciences*.

[26] Ortiz GG, Elena Crespo-López M, Morán-Moguel C, García JJ, Reiter RJ, Acuña-Castroviejo D (2001). Protective role of melatonin against MPTP-induced mouse brain cell DNA fragmentation and apoptosis in vivo. *Neuroendocrinology Letters*.

[27] Paxinos G, Watson C (1986). *The Rat Brain in Stereotaxic Coordinates*.

[28] Ungerstedt U, Arbuthnott GW (1970). Quantitative recording of rotational behavior in rats after 6-hydroxy-dopamine lesions of the nigrostriatal dopamine system. *Brain Research*.

[29] Cenci MA, Lee CS, Björklund A (1998). L-DOPA-induced dyskinesia in the rat is associated with striatal overexpression of prodynorphin- and glutamic acid decarboxylase mRNA. *European Journal of Neuroscience*.

[30] Lee CS, Cenci MA, Schulzer M, Björklund A (2000). Embryonic ventral mesencephalic grafts improve levodopa-induced dyskinesia in a rat model of Parkinson’s disease. *Brain*.

[31] Lundblad M, Andersson M, Winkler C, Kirik D, Wierup N, Cenci Nilsson MA (2002). Pharmacological validation of behavioural measures of akinesia and dyskinesia in a rat model of Parkinson’s disease. *European Journal of Neuroscience*.

[32] Avila-Costa MR, Montiel Flores E, Colin-Barenque L (2004). Nigrostriatal modifications after vanadium inhalation: an immunocytochemical and cytological approach. *Neurochemical Research*.

[33] Avila-Costa MR, Colín-Barenque L, Montiel-Flores E (2005). Bromocriptine treatment in a Murine Parkinson’s model: ultrastructural evaluation after dopaminergic deafferentation. *International Journal of Neuroscience*.

[34] Avila-Costa MR, Colín-Barenque L, Aley-Medina P (2005). Bilateral increase of perforated synapses after unilateral dopamine depletion. *International Journal of Neuroscience*.

[35] Calverley RKS, Jones DG (1987). A serial-section study of perforated synapses in rat neocortex. *Cell and Tissue Research*.

[36] Nutt JG (1990). Levodopa-induced dyskinesia: review, observations, and speculations. *Neurology*.

[37] Fahn S (2000). The spectrum of levodopa-induced dyskinesias. *Annals of Neurology*.

[38] Allbutt HN, Henderson JM (2007). Use of the narrow beam test in the rat, 6-hydroxydopamine model of Parkinson’s disease. *Journal of Neuroscience Methods*.

[39] Truong L, Allbutt H, Kassiou M, Henderson JM (2006). Developing a preclinical model of Parkinson’s disease: a study of behaviour in rats with graded 6-OHDA lesions. *Behavioural Brain Research*.

[40] Blanchet PJ, Boucher R, Bedard PJ (1994). Excitotoxic lateral pallidotomy does not relieve L-DOPA-induced dyskinesia in MPTP parkinsonian monkeys. *Brain Research*.

[41] Rajput AH, Fenton ME, Di Paolo T, Sitte H, Pifl C, Hornykiewicz O (2004). Human brain dopamine metabolism in levodopa-induced dyskinesia and wearing-off. *Parkinsonism and Related Disorders*.

[42] Mercuri NB, Bernardi G (2005). The ‘magic’ of L-dopa: why is it the gold standard Parkinson’s disease therapy?. *Trends in Pharmacological Sciences*.

[43] Blanchet PJ, Konitsiotis S, Whittemore ER, Zhou ZL, Woodward RM, Chase TN (1999). Differing effects of N-methyl-D-aspartate receptor subtype selective antagonists on dyskinesias in levodopa-treated 1-methyl-4-phenyl- tetrahydropyridine monkeys. *Journal of Pharmacology and Experimental Therapeutics*.

[44] Linazasoro G (2005). New ideas on the origin of L-dopa-induced dyskinesias: age, genes and neural plasticity. *Trends in Pharmacological Sciences*.

[45] Samadi P, Bédard PJ, Rouillard C (2006). Opioids and motor complications in Parkinson’s disease. *Trends in Pharmacological Sciences*.

[46] Jaber M, Dumartin B, Saund C (1999). Diferential regulation of tyrosine hydroxilase in the ganglia basal of mice lacking the dopamine transporter. *European Journal of Neuroscience*.

[47] Fahn S, Oakes D, Shoulson I (2004). Levodopa and the progression of Parkinson’s disease. *The New England Journal of Medicine*.

[48] Kearns CM, Gash DM (1995). GDNF protects nigral dopamine neurons against 6-hydroxydopamine in vivo. *Brain Research*.

[49] Kumar R, Agarwal AK, Seth PK (1995). Free radical-generated neurotoxicity of 6-hydroxydopamine. *Journal of Neurochemistry*.

[50] Tomás-Zapico C, Coto-Montes A (2005). A proposed mechanism to explain the stimulatory effect of melatonin on antioxidative enzymes. *Journal of Pineal Research*.

[51] Singh S, Ahmed R, Sagar RK, Krishana B (2006). Neuroprotection of the nigrostriatal dopaminergic neurons by melatonin in hemiparkinsonium rat. *Indian Journal of Medical Research*.

[52] Hamdi A (1998). Melatonin administration increases the affinity of D2 dopamine receptors in the rat striatum. *Life Sciences*.

[53] Aguiar LMV, Vasconcelos SMM, Sousa FCF, Viana GSB (2002). Melatonin reverses neurochemical alterations induced by 6-OHDA in rat striatum. *Life Sciences*.

[54] Damier P, Hirsch EC, Agid Y, Graybiel AM (1999). The substantia nigra of the human brain: II. Patterns of loss of dopamine-containing neurons in Parkinson’s disease. *Brain*.

[55] Dowd E, Dunnett SB (2005). Comparison of 6-hydroxydopamine-induced medial forebrain bundle and nigrostriatal terminal lesions in a lateralised nose-poking task in rats. *Behavioural Brain Research*.

[56] Picconi B, Centonze D, Rossi S, Bernardi G, Calabresi P (2004). Therapeutic doses of L-dopa reverse hypersensitivity of corticostriatal D2-dopamine receptors and glutamatergic overactivity in experimental parkinsonism. *Brain*.

[57] Luo Y, Roth GS (2000). The roles of dopamine oxidative stress and dopamine receptor signaling in aging and age-related neurodegeneration. *Antioxidants and Redox Signaling*.

[58] Greenamyre JT, Hastings TG (2004). Parkinsons-divergent causes convergent mechanisms. *Science*.

[59] Glinka Y, Gassen M, Youdim MBH (1997). Mechanism of 6-hydroxydopamine neurotoxicity. *Journal of Neural Transmission, Supplement*.

[60] Antolín I, Mayo JC, Sainz RM (2002). Protective effect of melatonin in a chronic experimental model of Parkinson’s disease. *Brain Research*.

[61] Acuña-Castroviejo D, Martín M, Macías M (2001). Melatonin, mitochondria, and cellular bioenergetics. *Journal of Pineal Research*.

[62] Dabbeni-Sala FSDS, Franceschini D, Skaper SD, Giusti P (2001). Melatonin protects against 6-OHDA-induced neurotoxicity in rats: a role for mitochondrial complex I activity. *The FASEB Journal*.

[63] Joo WS, Jin BK, Park CW, Maeng SH, Kim YS (1998). Melatonin increases striatal dopaminergic function in 6-OHDA-lesioned rats. *NeuroReport*.

[64] Kim YS, Joo WS, Jin BK, Cho YH, Baik HH, Park CW (1998). Melatonin protects 6-OHDA-induced neuronal death of nigrostriatal dopaminergic system. *NeuroReport*.

[65] Jin BK, Shin DY, Jeong MY (1998). Melatonin protects nigral dopaminergic neurons from 1-methyl-4- phenylpyridinium (MPP+) neurotoxicity in rats. *Neuroscience Letters*.

[66] Cotzias GC, Tang LC, Miller ST, Ginos JZ (1971). Melatonin and abnormal movements induced by L-dopa in mice. *Science*.

[67] Venero JL, Absi EH, Cano J, Machado A (2002). Melatonin induces tyrosine hydroxylase mRNA expression in the ventral mesencephalon but not in the hypothalamus. *Journal of Pineal Research*.

[68] Ingham CA, Hood SH, Arbuthnott GW (1991). A light and electron microscopical study of enkephalin-immunoreactive structures in the rat neostriatum after removal of the nigrostriatal dopaminergic pathway. *Neuroscience*.

[69] Pickel VM, Johnson E, Carson M, Chan J (1992). Ultrastructure of spared dopamine terminals in caudate-putamen nuclei of adult rats neonatally treated with intranigral 6-hydroxydopamine. *Developmental Brain Research*.

[70] Avila-Costa M, Gutierrez-Valdez A, Ordoñez-Librado J (2008). Time course changes of the striatum neuropil after unilateral dopamine depletion and the usefulness of the contralateral striatum as a control structure. *Neurological Research*.

[71] Bogaerts V, Theuns J, Van Broeckhoven C (2008). Genetic findings in Parkinson’s disease and translation into treatment: a leading role for mitochondria?. *Genes, Brain and Behavior*.

[72] Schapira AH (2008). Mitochondria in the aetiology and pathogenesis of Parkinson’s disease. *The Lancet Neurology*.

[73] Crespo E, Macías M, Pozo D (1999). Melatonin inhibits expression of the inducible NO synthase II in liver and lung and prevents endotoxemia in lipopolysaccharide-induced multiple organ dysfunction syndrome in rats. *The FASEB Journal*.

[74] Escames G, León J, Macías M, Khaldy H, Acuña-Castroviejo D (2003). Melatonin counteracts lipopolysaccharide-induced expression and activity of mitochondrial nitric oxide synthase in rats. *The FASEB Journal*.

[75] López LC, Escames G, Tapias V, Utrilla P, León J, Acuña-Castroviejo D (2006). Identification of an inducible nitric oxide synthase in diaphragm mitochondria from septic mice: its relation with mitochondrial dysfunction and prevention by melatonin. *International Journal of Biochemistry and Cell Biology*.

[76] Tapias V, Escames G, López LC (2009). Melatonin and its brain metabolite N1-acetyl-5-methoxykynuramine prevent mitochondrial nitric oxide synthase induction in Parkinsonian mice. *Journal of Neuroscience Research*.

[77] Martín M, Macías M, Escames G, León J, Acuña-Castroviejo D (2000). Melatonin but not vitamins C and E maintains glutathione homeostasis in t-butyl hydroperoxide-induced mitochondrial oxidative stress. *The FASEB Journal*.

[78] Roberts RC, DiFiglia M (1990). Evidence for synaptic proliferation, reorganization, and growth in the excitotoxic lesioned adult rat caudate nucleus. *Experimental Neurology*.

[79] Stephens B, Mueller AJ, Shering AF (2005). Evidence of a breakdown of corticostriatal connections in Parkinson’s disease. *Neuroscience*.

[80] Forno LS, Norville RL (1979). Ultrastructure of the neostriatum Huntington’s and Parkinson’s disease. *Advances in Neurology*.

[81] McNeill TH, Brown SA, Rafols JA, Shoulson I (1988). Atrophy of medium spiny I striatal dendrites in advanced Parkinson’s disease. *Brain Research*.

[82] Machado-Salas J, Ibarra O, Martinez Fong D, Cornejo A, Aceves J, Kuri J (1990). Degenerative ultrastructural changes observed in the neuropil of caudate nuclei from Parkinson’s disease patients. *Stereotactic and Functional Neurosurgery*.

[83] Schuster S, Doudnikoff E, Rylander D (2009). Antagonizing L-type Ca2+ channel reduces development of abnormal involuntary movement in the rat model of L-3,4-dihydroxyphenylalanine-induced dyskinesia. *Biological Psychiatry*.

[84] Neely MD, Schmidt DE, Deutch AY (2007). Cortical regulation of dopamine depletion-induced dendritic spine loss in striatal medium spiny neurons. *Neuroscience*.

[85] Shen W, Tian X, Day M (2007). Cholinergic modulation of Kir2 channels selectively elevates dendritic excitability in striatopallidal neurons. *Nature Neuroscience*.

[86] Freund TF, Powell JF, Smith AD (1984). Tyrosine hydroxylase-immunoreactive boutons in synaptic contact with identified striatonigral neurons, with particular reference to dendritic spines. *Neuroscience*.

[87] Zahm DS (1992). An electron microscopic morphometric comparison of tyrosine hydroxylase immunoreactive innervation in the neostriatum and the nucleus accumbens core and shell. *Brain Research*.

[88] Groves PM, Linder JC, Young SJ (1994). 5-Hydroxydopamine-labeled dopaminergic axons: three-dimensional reconstructions of axons, synapses and postsynaptic targets in rat neostriatum. *Neuroscience*.

[89] Anglade P, Mouatt-Prigent A, Agid Y, Hirsch EC (1996). Synaptic plasticity in the caudate nucleus of patients with Parkinson’s disease. *Neurodegeneration*.

[90] Hanley JJ, Bolam JP (1997). Synaptology of the nigrostriatal projection in relation to the compartmental organization of the neostriatum in the rat. *Neuroscience*.

[91] Ingham CA, Hood SH, Taggart P, Arbuthnott GW (1998). Plasticity of synapses in the rat neostriatum after unilateral lesion of the nigrostriatal dopaminergic pathway. *Journal of Neuroscience*.

[92] Ingham CA, Hood SH, Taggart P, Arbuthnott GW, Ohye C, Kimura M, McKenzie JS (1996). Synaptic plasticity in the rat neostriatum after unilateral 6-hydroxydopaminelesion of the nigrostriatal dopaminergic pathway. *The Basal Ganglia*.

[93] Reynolds JNJ, Hyland BI, Wickens JR (2001). A cellular mechanism of reward-related learning. *Nature*.

[94] Picconi B (2001). Effects of dopamine denervation and chronic levodopa treatment of synaptic plasticity and spontaneous synaptic activity of stratal spiny neurones. *Society for Neuroscience Abstract*.

[95] Calabresi P, Giacomini P, Centonze D, Bernardi G (2000). Levodopa-induced dyskinesia: a pathological form of striatal synaptic plasticity?. *Annals of Neurology*.

[96] Centonze D, Picconi B, Gubellini P, Bernardi G, Calabresi P (2001). Dopaminergic control of synaptic plasticity in the dorsal striatum. *European Journal of Neuroscience*.

[97] Parish CL, Finkelstein DI, Drago J, Borrelli E, Horne MK (2001). The role of dopamine receptors in regulating the size of axonal arbors. *Journal of Neuroscience*.

[98] Meshul CK, Tan SE (1994). Haloperidol-induced morphological alterations are associated with changes in calcium/calmodulin kinase II activity and glutamate immunoreactivity. *Synapse*.

[99] Meredith GE, De Souza IEJ, Hyde TM, Tipper G, Mai Luen Wong, Egan MF (2000). Persistent alterations in dendrites, spines, and dynorphinergic synapses in the nucleus accumbens shell of rats with neuroleptic-induced dyskinesias. *Journal of Neuroscience*.

[100] Meshul CK, Allen C (2000). Haloperidol reverses the changes striatal glutamatergic immunolabeling following a 6-OHDA lesion. *Synapse*.

[101] Geinisman Y, Morrell F, DeToledo-Morrell L (1992). Increase in the number of axospinous synapses with segmented postsynaptic densities following hippocampal kindling. *Brain Research*.

[102] Nieto-Sampedro M, Hoff SF, Cotman CW (1982). Perforated postsynaptic densities: probable intermediates in synapse turnover. *Proceedings of the National Academy of Sciences of the United States of America*.

[103] Carlin RK, Siekevitz P (1983). Plasticity in the central nervous system: do synapses divide?. *Proceedings of the National Academy of Sciences of the United States of America*.

[104] Geinisman Y, De Toledo-Morrell L, Morrell F (1986). Aged rats need a preserved complement of perforated axospinous synapses per hippocampal neuron to maintain good spatial memory. *Brain Research*.

[105] See RE, Chapman MA, Menshul CK (1992). Comparison of chronic intermit- tent haloperidol and raclopride effects on striatal dopamine release and synaptic ultra- structure in rats. *Synapse*.

[106] Ungerstedt U (1971). Postsynaptic supersensitivity after 6-hydroxy-dopamine induced degeneration of the nigro-striatal dopamine system. *Acta Physiologica Scandinavica, Supplement*.

[107] Avila-Costa MR, Gutierrez-Valdez AL, Ordoñez-Librado JL, Kaiser TF, Peters FJ (2008). The presence of perforated synapses in the striatum after dopamine depletion. Is this a sign of Negative Brain Plasticity?. *Synaptic Plasticity: New Research*.

